# Incorporating clinicopathological and molecular risk prediction tools to improve outcomes in early HR+/HER2– breast cancer

**DOI:** 10.1038/s41523-023-00560-z

**Published:** 2023-06-28

**Authors:** Giuseppe Curigliano, Rebecca Dent, Antonio Llombart-Cussac, Mark Pegram, Lajos Pusztai, Nicholas Turner, Giuseppe Viale

**Affiliations:** 1 European Institute of Oncology, IRCCS, Milan, Italy; 2 Department of Oncology and Hemato-Oncology, University of Milano, Milan, Italy; 3 National Cancer Centre, Singapore, Singapore; 4 University Hospital Arnau de Vilanova, Valencia, Spain; 5 Stanford University, Palo Alto, CA USA; 6 Yale School of Medicine, New Haven, CT USA; 7 Institute of Cancer Research, London, UK

**Keywords:** Prognostic markers, Risk factors

## Abstract

Stratification of recurrence risk is a cornerstone of early breast cancer diagnosis that informs a patient’s optimal treatment pathway. Several tools exist that combine clinicopathological and molecular information, including multigene assays, which can estimate risk of recurrence and quantify the potential benefit of different adjuvant treatment modalities. While the tools endorsed by treatment guidelines are supported by level I and II evidence and provide similar prognostic accuracy at the population level, they can yield discordant risk prediction at the individual patient level. This review examines the evidence for these tools in clinical practice and offers a perspective of potential future risk stratification strategies. Experience from clinical trials with cyclin D kinase 4/6 (CDK4/6) inhibitors in the setting of hormone receptor–positive (HR+)/human epidermal growth factor receptor 2-negative (HER2-) early breast cancer is provided as an illustrative example of risk stratification.

## Introduction

Overall, approximately 12–15% of people with stage I–III HR+ breast cancer will experience metastatic recurrence^1^. However, risk is not distributed evenly across clinical stage, and in addition to tumor size and nodal status, several other variables influence prognosis and several of these (e.g., histologic grade, the Oncotype DX^®^ 21-gene recurrence score) are now incorporated into the 8th edition of the American Joint Committee on Cancer (AJCC) staging guidelines^2^. Accurate prediction of the absolute risk of local or distant recurrence that a patient faces after locoregional therapy is important for judging the risk-benefit ratio of systemic therapies. One of the challenges inherent with managing HR+ early breast cancer—and HR+/HER2- cancer in particular—is the persistent risk of recurrence that extends over decades, with as much as 50% of recurrences occurring more than 5 years after diagnosis^3^. Indeed, late recurrence (i.e., after 5 years of follow-up) risk prediction tools are emerging that are increasingly used to assist decision-making about extended adjuvant endocrine therapy (ET)^4^.

Breast tumors are highly heterogeneous with differences in morphology and molecular features, and many known and unknown factors influence the likelihood of response to treatment and risk of recurrence^3,5^. Multigene molecular tests capture some of these molecular features (most consistently proliferation and estrogen receptor [ER] signaling) and integrate them into a single risk score^6–10^. These assays can provide an estimate of the probability of recurrence as well as assign a risk category (low, high, intermediate); however, the most accurate risk prediction requires integration of tumor size, nodal status, grade, and patient age/menopausal status with the multigene risk scores^11^. Lymphovascular invasion is an additional prognostic factor that has significant negative impact on survival in N0 disease^12^. Since each multigene assay uses different sets of genes and weighs components differently, the individual patient level risk predictions can be discordant, particularly for patients who are in the intermediate-risk category^3,13^. In a direct comparison of several assays, including the Oncotype DX 21-gene recurrence score, MammaPrint, and PAM50 risk of recurrence score (Prosigna^®^), in the OPTIMA prelim trial, fewer than 40% of tumors were classified similarly by all tests^13^. Similarly, a review comparing risk classifications of the Oncotype DX 21-gene recurrence score, Breast Cancer Index, EndoPredict, MammaPrint, and Prosigna assays demonstrated distribution discrepancies among risk groups^14^.

This review examines the currently available clinical and transcriptomic tools for risk stratification, current evidence to support their use in different patient populations, and the extent to which they can inform treatment decisions. We will conclude by briefly discussing the future of risk stratification and generation of predictive tools to guide treatment decisions.

## Results

### Clinicopathologic risk assessment

Following diagnosis of breast cancer, histopathologic and molecular assessment have long been the cornerstones of risk stratification^15^. The AJCC guidelines recommend anatomic staging based on tumor size, and the existence of lymphadenopathy and distant metastases, histological tumor grade, and expression of ER, progesterone receptor (PR), HER2, and Ki-67^16^. Currently, PREDICT is one of the most widely available clinically validated prognostic risk assessment tools that uses information from routinely available clinical and pathologic variables (https://breast.predict.nhs.uk/tool)^17,18^. Clinical Treatment Score post-5 years (CTS5) is another commonly used tool; it was specifically developed to predict risk of recurrence between years 5 and 10 for women with ER+ breast cancer who are recurrence-free 5 years after ET. The CTS5 calculator is freely available online at https://www.cts5-calculator.com and combines information from tumor size, grade, age, and number of nodes involved^19^. Ki-67-related antigen is a clinicopathologic marker of cellular proliferation that can be detected using immunohistochemistry^20^. Its expression has been associated with breast cancer prognosis, including in early breast cancer^20–22^. Ki-67 is predictive of response to neoadjuvant ET^23,24^. Furthermore, suppression of Ki-67 expression in the setting of preoperative ET is prognostic for recurrence-free survival^23,25^. However, until recently, Ki-67 has not been included in routine clinical decision-making as its relevance to known targets has been unclear and hindered by concerns such as inter-laboratory variability and lack of cut-off consensus^26,27^. Another commonly used prognostic index is the preoperative endocrine prognostic index (PEPI) which utilizes pathological tumor size, node status, Ki-67 labeling index, and ER status following neoadjuvant therapy^28^. Unfortunately, classification based on clinicopathologic assessment alone may not sufficiently capture a patient’s prognosis, especially for cases where decisions are challenging^3,29^. More recently, gene expression profiling (including genomic subtypes luminal A, luminal B, luminal HER2, HER2-enriched, basal-like, and triple negative), as well as multigene panels have allowed for more nuanced prognostic profiling^16^.

### Transcriptomic risk stratification tools

There are a number of commercially available and clinically validated transcriptomic risk stratification tools, each of which assesses a different suite of genes to generate a prognostic score (Table 1).

**Table 1:** Tumor genomic assay used in breast cancer to determine recurrence risk in prospective randomized trials.

	NUMBER OF GENES	PROFILING TECHNIQUE	RISK STRATIFICATION	VALIDATION STUDIES	OUTCOMES	
ONCOTYPE DX	21 genes (16 genes + 5 reference)	RT-PCR	Recurrence Score (RS; range 0–100) Low < 18 Int 18–30 High > 30	NSABP B14 668 pts, ER+, N0, treated with TAM	10-years DR Low (51%): 6.8% Int (22%): 14.3% High (27%): 30.5%	
				NSABP B20 651 pts, ER+, N0, treated with TAM (227) or TAM+CT (424)	10-years DRF Low (54%): 97% (w/o CT), 96% (w CT) Int (21%): 91% (w/o CT), 89% (w CT) High (25%): 60% (w/o CT), 88% (w CT)	
				SWOG 8814 367 pts, postmenopausal, ER+, N+, treated with TAM (148) or TAM+CT (219)	10-years DFS Low (40%): 60% (w/o CT), 64% (w CT) Int (28%): 49% (w/o CT), NR (w CT) High (32%): 43% (w/o CT), 55% (w CT)	
				TransATAC 1231 pts, postmenopausal, ER+, N0 (872) or N+ (306), treated with TAM or ANA	9-years DR Low: N0 4%; N+17% Int: N0 12%; N+28% High: N0 25%; N+49%	
			Low < 11 Int 11–25 High > 25	TAILORx 10253 pts, ER+/HER2-, N0. Low risk (17%) received ET only, High-risk (14%) received ET+CT, Int-risk (69%) randomized to ET only or ET+CT	9-years IDFS Low: 84% Int: 83% (w/o CT), 84% (w CT) High: 76%	9-years 0 S Low: 94% Int: 94% (w/o CT), 94% (w CT) High: 89%
				RxPonder 5083 pts, HR+/HER2-, N1 (1–3 positive nodes), RS< 25, randomized to ET alone or ET+CT	5-years IDFS Premenopausal: 93.9% (w CT) 89.0 % (w/o CT) Postmenopausal: 91.9% (w CT) 91.3 % (w/o CT)	5-years OS Premenopausal: 98.6% (w CT) 97.3 % (w/o CT) Postmenopausal: 96.2% (w CT) 96.1 % (w/o CT)
MAMMAPRINT	70 genes	DNA microarray	MammaPrint Index (MPI; range −1; +1) Low risk (1.0; 0) High risk (0; +1.0)	TRANSBIG 302 pts, <61 years, T1-T2, N0, ER+ (212) and ER- (90), all untreated with any adjuvant systemic therapy	10-years OS G-high risk: 69% (C-low risk), 69% (C-high risk) G-low risk: 89% (C-high risk), 88% (C-low risk)	
				RASTER 427 pts, cT1–3, N0, < 61 years, ER+ (80%) and ER- (20%), HER2+ (11%) and HER2- (84%). 202 pts received CT (15% of G-low and 88% of G-high), 168 pts did not receive any adj systemic therapy	5-years DRFI G-low/C-low (95): 95% G-high/C-low (37): 100% G-low/C-high (124): 98% G-high/C-high (171): 90%	5-years DDFS G-low/C-low (95): 94% G-high/C-low (37): 95% G-low/C-high (124): 98% G-high/C-high (171): 89%
				MINDACT 6693 pts, T1-2 or operable T3, N0 (79%) or N1 (21%); HER2+ (10%) or HER2- (90%) 4 subgroups: G-low/C-low (41%), receiving ET G-high/C-low (8.8%): receiving ET or ET+CT G-low/C-high (23.2%): receiving ET or ET+CT G-high/C-high (27%): receiving ET+CT	5-years DMFS G-low/C-low: 97.6% (w/o CT) G-high/C-low: 94.8% (95.8% w CT; 95.0% w/o CT) G-low/C-high: 95.1% (95.9% w CT; 94.4% w/o CT) G-high/C-high: 90.6% (w CT)	

If not specified, both pre- and post-menopausal patients were included. *DFS* Disease-free survival, *DMFS* Distant metastasis-free survival, *DR* Distant recurrence, *DRF* Distant recurrence-free, *DRFI* Distant relapse-free interval, *IDFS* Invasive disease-free survival, *MFS* Metastasis-free survival; OS: overall survival.

#### Oncotype DX

The most widely recommended genomic test is Oncotype DX Breast Recurrence Score^®^ Test, often referred to as the 21-gene recurrence score assay. Oncotype DX uses gene expression data to calculate a recurrence score (RS) from 0 to 100 and provides a percent risk of distant recurrence over the next 9 years with ET alone and estimates the predicted benefit from adjuvant chemotherapy^30,31^. Estimates are adjusted for nodal status (positive vs. negative) and the company provides a free web tool (RSClin: https://online.genomichealth.com), which integrates RS with tumor size and grade to improve prognostic accuracy^32^. RSClin integrates the 21-gene RS with tumor grade, tumor size, and age. It was developed using data from a patient-specific meta-analysis of 10,004 women with HR + /HER2-, node-negative breast cancer. This database included 577 women from the NSABP B-14 study who received ET alone^33^, 4854 from TAILORx who received ET alone, and 4573 who also received chemotherapy^34^. Oncotype DX has been prospectively validated for pre- and postmenopausal patients with HR + /HER2- node-negative, and node-positive (1–3 positive nodes) disease^35^.

#### Mammaprint

MammaPrint is a similar transcriptomic assay that was also validated in a prospective trial and in multiple prospectively designed retrospective studies. It uses information from 70 genes to assign a MammaPrint low- or high-risk category^10,36^. The prognostic accuracy of MammaPrint is best established in node-negative patients due to the relatively small number of patients with positive nodes who were included in prospective trials with this assay^10,29,36,37^. The 70-gene signature can identify patients with an ultra-low risk of distant recurrence. Of the 6693 patients enrolled in the MINDACT trial, profiling revealed an ultra–low-risk 70-gene signature in 1000 patients. After a median follow-up of 8.7 years, 8-year distant metastasis-free interval in these patients was 97.0% vs. 94.5% for patients with low-risk signature and 89.2% for patients with high-risk signature^38^.

#### Prosigna and endopredict^®^

Two transcriptomic assays, Prosigna and EndoPredict^®^, incorporate clinical information in addition to molecular measurements in the algorithm for generating a prognostic risk score^8^. Prosigna is based on the PAM50 assay, incorporating tumor size and an estimate of molecular class (e.g., basal-like, HER2-enriched, luminal A, luminal B) to separate patients into low-, intermediate-, and high-risk groups based on a score^8^. EndoPredict combines transcriptomic and clinical risk factors (e.g., nodal status and tumor size) to categorize patients into high or low risk of recurrence groups^39^. EndoPredict is validated for use in postmenopausal patients with node-positive or node-negative disease, but validation is lacking for premenopausal patients^39,40^.

#### Breast cancer index

The Breast Cancer Index (BCI) risk of recurrence and extended endocrine benefit test is another multigene assay endorsed by guidelines for women with lymph node–negative or lymph node–positive disease^29,41^. The test provides a quantitative estimate of overall risk of recurrence over 10 years and a separate estimate for late distant recurrence (i.e., after 5 years)^30,36^. Uniquely, among all other transcriptomic assays, the BCI also provides a prediction of the likelihood of benefit from extended (greater than 5 years) adjuvant ET^4^. It has been validated in several prospectively designed retrospective studies, though not all validation studies have confirmed its predictive value for extended adjuvant ET benefit, due at least in part to a lack of statistical power^4,42–45^.

### Role of risk stratification tools in informing treatment decisions for HR+/HER2- disease

#### RS group considerations

Some women with HR+/HER2- early breast cancer will gain substantial benefit from adjuvant (or neoadjuvant) chemotherapy, whereas many other patients can safely avoid chemotherapy. Over the past 15 years, important progress has been made in identifying the low-risk population who can safely forego adjuvant chemotherapy. Prior to the prospective analysis performed in the TAILORx study^46^, early work from the National Surgical Adjuvant Breast and Bowel Project (NSABP) B20 trial classified patients into low-, intermediate-, and high-risk groups based on Oncotype DX RS of <18, 18– < 31, and ≥ 31, respectively^47^. A retrospective analysis of the trial showed that patients with Oncotype DX RS of < 18 had excellent long-term outcomes and derived no apparent benefit from combination chemotherapy plus tamoxifen treatment vs. tamoxifen^47^. Similarly, patients with RS of 18–30 did not appear to receive substantial benefit from chemotherapy plus tamoxifen treatment vs. tamoxifen^47^. On the other hand, patients with RS ≥ 31 had a much higher absolute risk of recurrence and experienced a 27.6% decrease in absolute risk of distant recurrence with chemotherapy plus tamoxifen vs. tamoxifen^47^. Predictive potential has also been suggested for MammaPrint and for neoadjuvant ET for EndoPredict^48,49^. Additionally, a PEPI score of 0 after neoadjuvant ET is associated with a low risk of relapse without chemotherapy^28^. These findings, along with other smaller studies, led to treatment guidelines that do not recommend administration of chemotherapy for patients with very low-risk HR + /HER2- early breast cancer, and endorse use of adjuvant chemotherapy for the high-risk group^29,41,50^.

While the clinical benefits of chemotherapy have been established for patients with high risk of recurrence, until very recently it remained unclear whether those with intermediate-risk scores derive benefit from chemotherapy. Data from the TAILORx trial, consisting of 6907 node-negative patients who had an Oncotype DX RS of 11–25, showed that ET was noninferior to adjuvant chemotherapy plus ET for invasive disease-free survival (iDFS), freedom from local or distant recurrence, and overall survival (hazard ratio: 1.08, *P* = 0.26; hazard ratio: 1.11, *P* = 0.33; hazard ratio: 0.99, *P* = 0.89, respectively)^7^. An exploratory analysis of the TAILORx trial suggested that women younger than 50 years with an intermediate-risk score had higher risk of recurrence than older women and derived a significant survival benefit from chemotherapy. This effect was particularly apparent between RS 21 and 25^7^. Whether this benefit is driven by the cytotoxic effect of the chemotherapy, or due to chemotherapy-induced amenorrhea, or both, remains unknown.

#### RS group and menopausal status considerations

While these results were generated from an unplanned exploratory analysis, they are nevertheless highly consistent with several other small and large studies that suggest higher risk and greater adjuvant chemotherapy benefit among younger HR+ patients^51^. For example, although underpowered, the exploratory analysis of the MINDACT study also indicated clinical benefit of chemotherapy for women aged less than 50 years at high clinical risk and low genomic risk^52^. The 8-year distant metastases-free survival (DMFS) with chemotherapy plus ET in women ≤ 50 years was 93.6% (95% CI: 89.3%–96.3%) compared with 88.6% (95% CI: 83.5%–92.3%) for ET alone^52^. Additionally, results from the RxPonder trial that included patients with 1–3 positive lymph nodes and RS ≤ 25 indicated no improvement of iDFS when adjuvant chemotherapy was added to ET for postmenopausal patients (hazard ratio: 0.97, *P* = 0.81)^6^. However, a substantial benefit was seen with the addition of adjuvant chemotherapy in premenopausal women (hazard ratio: 0.53, *P* < 0.001)^6^.

#### Challenges: Discordant results

Where tests are readily accessible, and more than one transcriptomic assay is performed on the same tissue, discordance between test results poses a challenge for clinical practice. The OPTIMA preliminary study used five different clinically validated tools (Oncotype DX, MammaPrint, Prosigna, IHC4, and IHC4-AQUA) to compare risk stratification for women aged ≥ 40 years with HR + /HER2- stage II–III breast cancer^13^. The study found substantial patient-level disagreement between the tests: overall, 60.6% of tumors were given different risk categories by at least one assay^13^. However, comparisons across tests are challenging because different tests use different thresholds to define high risk. Additionally, some assays provide only two risk categories (high vs. low), while others provide three categories (low, intermediate, high)^6–10^. The tests also use different genes to calculate risk; for example, while MammaPrint and Prosigna quantify 70 and 50 genes, respectively, only three of these are common between the two tests^13^.

Based on currently available data, it is not possible to determine whether one test is overall superior to others, or if one test is more suited to a particular patient population. For this reason, guidelines discourage use of multiple transcriptomic tests, and clinicians should avoid redundant testing, as instead of improved precision, it leads to greater confusion^37^.

#### Use of tests in resource-constrained settings

Despite the cost-effectiveness and international guideline support for using transcriptomic tests^29,41,50,53^, testing is not routinely available for all patients in different parts of the world. The St. Gallen International Consensus Guidelines recognize that while the use of transcriptomic assays is preferable for patients with intermediate risk, integration of traditional clinical factors (tumor grade, ER, PR, and HER2 status, and proliferation by Ki-67 assessment) can also be used to identify patients at low risk of recurrence to inform adjuvant chemotherapy treatment decisions when the more accurate and standardized transcriptomic assays are not available^50^.

### Risk stratification in clinical trials: Lessons from adjuvant studies of CDK4/6 inhibitors

Preliminary studies have found that CDK4/6 inhibitors abemaciclib, palbociclib, and ribociclib significantly lower Ki-67 expression^54–56^. As a result, this class of drugs may have a role in treatment of highly proliferative tumors in the early breast cancer setting. Indeed, these agents have demonstrated significantly improved progression-free survival and improved overall survival for some patients in metastatic HR + /HER2- breast cancer^54,57–61^.

**Fig. 1:**
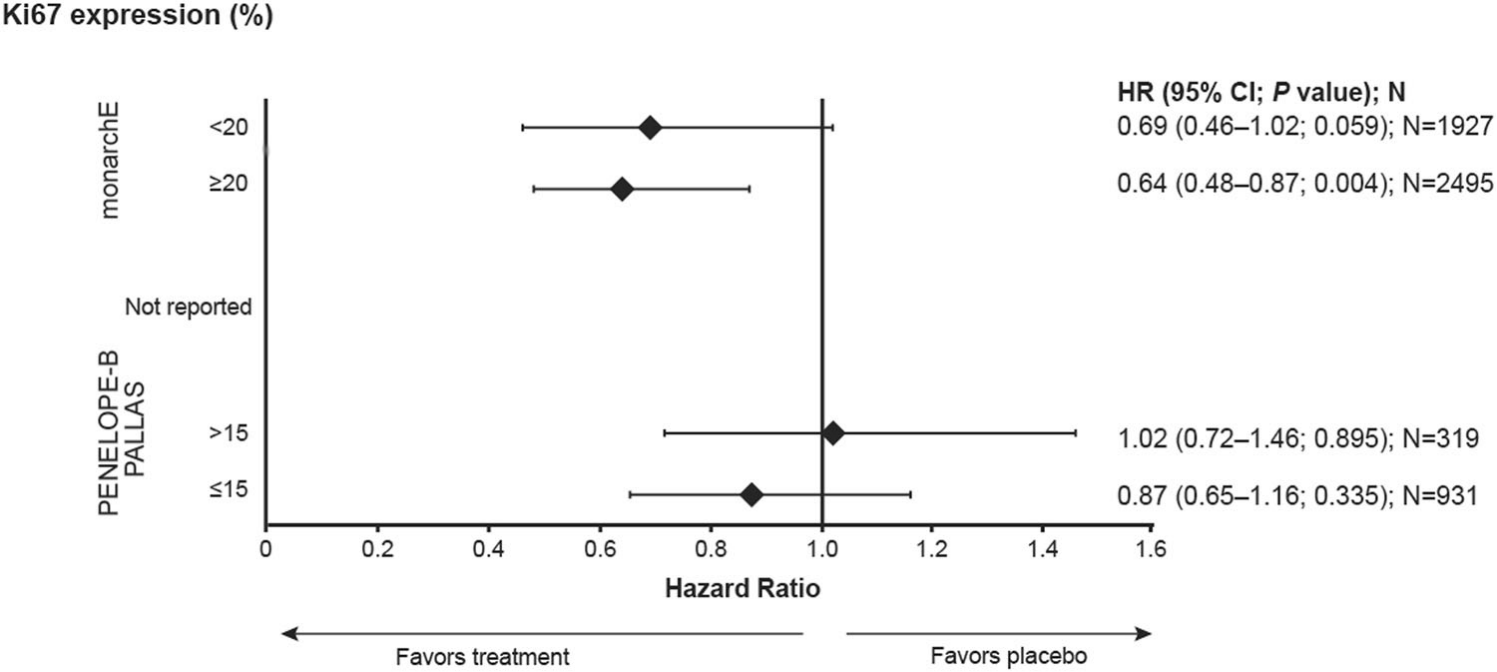
Impact of Ki-67 expression on iDFS following CDK4/6 inhibition^62–64^. Two-sided analysis. Error bars are representative of 95% CI. CDK4/6 Cyclin D kinase 4/6, HR Hazard ratio, iDFS Invasive disease-free survival.

A number of phase 3 clinical trials investigating this class of drugs in the adjuvant setting have recently been completed or are ongoing (Table 2). Recent data from the monarchE, PALLAS, and PENELOPE-B trials show differing results for the use of CDK4/6 inhibitors for HR + /HER2- early breast cancer^62–64^. The monarchE trial was specifically designed to enroll a high-risk patient group based on number of positive lymph nodes, tumor size, grade, and Ki-67 expression^64^. In this trial (median follow-up of 27 months), abemaciclib in combination with ET demonstrated a statistically significant improvement in iDFS (2-year iDFS rate: 92.3%) in the intent-to-treat population compared to ET alone (2-year iDFS rate: 89.3%), with a 29% reduction in the risk of developing invasive disease (nominal *P* = 0.001)^64^. In patients with high Ki-67 of ≥ 20% (N = 2498), abemaciclib + ET demonstrated statistically significant improvement in iDFS at the primary outcome analysis (hazard ratio: 0.64, [95% CI: 0.48–0.87], *P* = 0.0042), and an absolute benefit of 7.1% in the 3-year iDFS rates (Fig. 1). Based on the efficacy results in cohort 1 patients with high Ki-67 scores at the additional follow-up analysis, the US Food and Drug Administration approved abemaciclib in combination with ET for patients with HR + /HER2-, node-positive, early breast cancer at high risk of recurrence and a Ki-67 score of ≥ 20%^65^; ASCO and NCCN have also updated their guidelines to include use of adjuvant abemaciclib plus ET in patients with ER + /HER2- early breast cancer and a Ki-67 score ≥ 20%^32,64,66^. In contrast, the PALLAS trial was stopped for futility at a pre-planned interim analysis, having shown no significant improvement in iDFS with adjuvant ET plus palbociclib vs. adjuvant ET alone^67^. At final analysis, the PENELOPE-B trial also showed no significant difference in iDFS for patients with residual disease who received adjuvant ET plus palbociclib compared with adjuvant ET alone at 4-year median follow-up; interestingly, the trial showed transient benefit in the initial 2–3 years, which was not seen with longer follow-up^62^. Notably the populations in PALLAS and PENELOPE-B did not use Ki-67 as an entry criterium as in the monarchE trial; indeed, only 25.5% of patients in the PALLAS trial had Ki-67 > 15%^62^. However, in a subgroup analysis, Ki-67 > 15% was not associated with an improved outcome relative to lower expression (Fig. 1)^62^. The use of the 15% threshold is further complicated by the fact that the clinical utility of Ki-67 at expression levels between 10 and 20% is limited in ER+/HER2- early breast cancer^68^.

**Table 2:** Trials for CDK4/6 Inhibitors in HR*+*/HER2- Early Breast Cancer.

	PALLAS^85^	PENELOPE-B^86^	monarchE^87^	NATALEE^88^
Study Design				
Sponsor/Collaborator	ABCSG/AFT	GBG	Eli Lilly/NSABP	Novartis/TRIO
NCT#	NCT02513394	NCT01864746	NCT03155997	NCT03701334
Design	Phase 3	Phase 3	Phase 3	Phase 3
	Randomized	Randomized	Randomized	Randomized
	Open label	Placebo-controlled	Open label	Open label
Sample size	5796	1250	5637	5101
Treatment arms	Palbociclib 125 mg QD, 3/1 schedule (2 years) + SOC ET vs. SOC ET	Palbociclib 125 mg QD, 3/1 schedule (1 year) + SOC ET vs. Placebo QD, 3/1 schedule (1 year) + SOC ET	Abemaciclib 150 mg BID (2 years) + SOC ET vs. SOC ET	Ribociclib 400 mg QD, 3/1 schedule (3 years) + SOC ET vs. SOC ET
Target population	Stage II/III (Stage IIA capped at 1000 pts)	Residual disease and an increased risk of recurrence after neoadjuvant chemotherapy	High-risk disease defined as lymph node + plus one other risk factor	Stage II/III
Key inclusion criteria	• ≤ 12 months since initial pathologic diagnosis • ≤ 6 months from first dose of SOC adj. ET if started • Prior chemotherapy (CTx) allowed	• ≤ 16 weeks of neoadjuvant CTx including at least 6 weeks of taxane-containing regimen • Residual invasive disease in breast or LN post-neoadjuvant therapy • CPS-EG score^a^ ≥ 3 or 2 if nodal status at surgery is ypN+	• Pathologic lymph node involvement + at least one of the following: ▪ ≥ 4 (+) axillary LN ▪ ≥ 5 cm tumor ▪ Grade 3 ▪ Ki-67 ≥ 20% on untreated breast tissue by central analysis • ≤ 16 months since definitive surgery • ≤ 12 weeks of ET until randomization following last non-ET (surgery, CTx, or radiation) whichever is last • Prior CTx allowed	• Definitive breast surgery for the current malignancy with/without Rx • Prior CTx allowed

^a^The CPS+EG score estimates relapse probability on the basis of clinical and pathologic stage (CPS) and estrogen receptor status and histologic grade (EG). Scores range from 0 to 6, with higher scores indicating higher risk of relapse^89^. *ABCSG/AFT* Austrian Breast & Colorectal Cancer Study Group/Alliance Foundation Trials, *BID* Two times a day, *CPS-EG* Clinical-Pathologic Scoring System incorporating estrogen receptor status and nuclear grade, *CTx* Chemotherapy, *ET* Endocrine therapy, *GBG* Global Benefits Group, *HER2* Human epidermal growth factor receptor 2, *HR* Hormone receptor, *LN* Lymph node, *LPFV* Last patient, first visit, *NCT* National Clinical Trial, *NSABP* National Surgical Adjuvant Breast and Bowel Project, *QD* Every day, *SOC* Standard of care.

The differences in outcome between the three trials is unexpected, as all CDK4/6 inhibitors in mBC have similar efficacy^69^. Potential explanations for the differences in outcome between the trials include different target populations, different baseline risk distribution (especially in terms of Ki-67 expression), different duration of treatment and follow-up, and potentially alleged differences in molecular mechanisms of action of the drugs (Table 2). Awaited data from the NATALEE trial are anticipated to provide more information on the potential adjuvant use of CDK4/6 inhibitors. Given that trial designs for monarchE, PALLAS, and PENELOPE-B pre-dated the era of transcriptome testing for risk stratification, the clinical utility of transcriptomic risk recurrence tools has not been clearly established with CDK4/6 inhibitors in early breast cancer with intermediate or high risk of recurrence; as such, focus directed toward ongoing trials of CDK4/6 inhibitors may shed light on the matter^70–72^.

### Guideline recommendations

AJCC, the National Comprehensive Cancer Network (NCCN), American Society of Clinical Oncology (ASCO), European Society for Medical Oncology (ESMO), and St. Gallen treatment guidelines recommend the use of transcriptomic assays to provide additional information for anatomic, histologic, and molecular-based staging^2,29,37,41,50^. Some of these guidelines groups assign different levels of confidence to the specific tests they recommend (Table 3), noting that most of these tests were designed specifically for use in ER+ tumors.

**Table 3:** Guideline Recommendations for Use of Genomic Tests.

	AJCC 8^th^ Edition^16^	NCCN^37^	ASCO^41^	ESMO^29^	St Gallen^50^
Node negative	Oncotype DX	Oncotype DX^a^ Prosigna Endopredict Breast Cancer Index	Oncotype DX^b^ Mammaprint^b,c^ Prosigna^b^ Endopredict^d,e^ Breast Cancer Index^d^	Mammaprint Oncotype DX^c^ Prosigna^c^ Endopredict^c^ Breast Cancer Index^c^	Strongly endorses value of genomic assays, but does not discuss specific tests
Node positive (1–3 positive nodes)		Oncotype DX^a^ Mammaprint Prosigna Endopredict Breast Cancer Index	Oncotype DX^b^ Mammaprint^b,c^ Endopredict^d^ Breast Cancer Index^d^		

^a^Preferred for node negative and for node positive postmenopausal.

^b^Strong recommendation (ER+/HER2-/node negative only).

^c^For patients ≥ 50 years of age with high clinical risk breast cancer.

^d^Moderate recommendation (ER+/HER2-/node negative only).

^e^Postmenopausal patients only. ESMO notes that these tests were developed for use in ER+ patients only. *AJCC* American Joint Committee on Cancer, *ASCO* American Society of Clinical Oncology, *ER* Estrogen receptor, *ESMO* European Society for Medical Oncology, *HER2* Human epidermal growth factor receptor 2, *NCCN* National Comprehensive Cancer Network.

The NCCN guidelines recommend considering transcriptomic testing for all patients with invasive ductal or lobular tumors greater than 0.5 cm in diameter and no lymph node involvement, and for patients with 1–3-node-positive disease who are candidates for adjuvant chemotherapy^37^. The St. Gallen International Consensus Guidelines also endorse the value of transcriptomic assays for determining whether to recommend chemotherapy in T1/T2 N0 tumors, T3 N0 tumors, and TxN1 (1–3 positive lymph nodes)^50^. ASCO and ESMO also make specific recommendations (Table 3).

Overall, the Oncotype DX RS has the largest body of evidence from prospective clinical trials to guide its use in the clinic and is the preferred assay according to the NCCN guidelines and AJCC staging (Table 3)^37^. NCCN gives the most detailed recommendations for translating Oncotype DX RS and other risk scores to clinical action. For node-negative premenopausal HR + /HER2- patients, NCCN guidelines indicate that there is no additional benefit to be gained from initiating chemotherapy with an Oncotype DX RS < 15, but recommend considering chemotherapy before ET, or ovarian suppression, for RS of 16–25, and addition of chemotherapy for RS ≥ 26. For patients with 1–3 positive lymph nodes, NCCN recommends addition of chemotherapy with a RS ≥ 26. For RS < 26, the recommendation to add chemotherapy depends on the menopausal status of the patient. ASCO has provided similar recommendations based on the results of TAILORx^4–6,41^.

## Dicussion

### The future of risk stratification

There are several emerging technologies that could potentially have a major impact on risk stratification in the not-too-distant future. Systemic recurrence in the absence of any detectable disease after surgical resection of the primary tumor by definition implies that a very small number of disseminated cancer cells survived adjuvant treatment and give rise to recurrence years later. Several methods have been developed to detect minimal residual disease (MRD). The presence of circulating tumor cells (CTCs) in blood in early breast cancer have been detected after surgery but before adjuvant chemotherapy in 21.5% of patients, but this was only associated with a modest reduction in DFS (hazard ratio: 2.1). Although a greater reduction in DFS was seen with patients who had > 5 CTCs detected (hazard ratio: 4.5), the population was small, accounting for only 3% of patients in the study. A stronger association was observed between presence of CTCs and risk of recurrence for patients with high-risk, HR+ disease at 5 years after diagnosis (hazard ratio: 13.1). However, CTC assays have low sensitivity and a risk of false-positive results and are not widely used in clinical practice^73^.

The major technological advance was the development of highly sensitive and specific circulating tumor DNA (ctDNA) assays, which can detect and quantify free tumor-derived DNA in plasma (see Coakley et al.^73^ for an in-depth discussion). The presence of ctDNA after completing treatment is associated with a very high risk of future relapse compared with absence of ctDNA (hazard ratio: 3.1–43.4 across a range of tumor types)^73^. In breast cancer, the presence of ctDNA after neoadjuvant therapy has been shown to be a good predictive marker of future relapse^74–76^. Regular ctDNA monitoring can also detect molecular relapse 8–11 months before clinical or radiological relapse is detectable^73^. In the metastatic setting, ctDNA has been shown to be more sensitive than CTC analysis to detect early progression and shows concordance with protein biomarkers such as CA27.29^77,78^. Results from metastatic breast cancer have found that ctDNA detection of variants of *ESR1*, *TP53*, and *PIK3CA* were predictive of poor overall survival, and may help to identify which patients may benefit from certain treatments^79^. For example, patients with *PIK3CA* mutations may benefit from treatment with PI3K inhibitors such as alpelisib^79^. Other potential roles of ctDNA may include monitoring treatment response and early detection of disease progression, detection of minimal residual disease, and obtaining information on the tumor when a biopsy is not feasible^80,81^.

These studies demonstrated technical robustness and clinical validity of ctDNA testing, but none of them have addressed its clinical utility, whether or not patient outcome improves because of ctDNA testing, or if ctDNA testing can help to optimize therapy. Several other important questions also remain unanswered, including the true prevalence of ctDNA positivity in different prognostic risk groups, the dynamics of ctDNA during follow-up in the absence of intervention, the proportion of patients who already have asymptomatic but detectable metastatic disease at the time of detecting ctDNA positivity, and most importantly, whether or not early therapeutic intervention at a molecular relapse state can improve clinical outcome (particularly overall survival). In addition, sampling time points and techniques have varied across studies conducted to date, indicating that standardization is required to inform routine clinical use of ctDNA sampling^64^.

Another new technological advance is the detection of mRNA. This approach has the potential to replace immunohistochemistry and in situ hybridization for evaluation of key biomarkers, including ER, PR, HER2, and Ki-67^68^. The recently developed STRAT4 assay is able to simultaneously detect expression of *ESR1*, *PGR*, *ERBB2*, and *MKi67*. This automated assay has the potential to eliminate the intra- and inter-observer variability inherent to immunohistochemistry and in situ hybridization.

Treatment guidelines recommend the use of gene expression tools to inform adjuvant chemotherapy selection for patients with early-stage HR+/HER2- breast cancers, with intermediate risk defined using clinical and histopathologic methods^29,37,41,82–84^. For individual patients, the classification of risk may vary between transcriptomic tools, thus necessitating a greater understanding of factors influencing discordance between assays^13^. Efforts are also underway to assess risk with greater granularity—such as improved detection of MRD. The implication of RS < 26 for pre- and postmenopausal women was clearly described in the RxPonder trial^6^. With a median follow-up of 5.3 years, and when adjusted for menopausal status, continuous recurrence score, and treatment group, the interaction between the continuous recurrence score and treatment group was not significant (*P* = 0.35). Hence, in women with N1 breast cancer and a recurrence score value of 0 to 25, the recurrence score did not significantly predict any relative improvement to iDFS as a result of chemotherapy treatment. In the overall trial population, patients who received chemoendocrine therapy had a significantly longer period of iDFS versus those who received endocrine therapy alone. iDFS for all participants at 5 years was 91.6%; specifically, 92.2% for the chemoendocrine group compared to 91.0% for the endocrine-only group (*P* = 0.10 by the log-rank test). There was no significant difference in iDFS between treatment groups for postmenopausal women, with iDFS at 5 years estimated at 91.3% in the chemoendocrine group and 91.9% in the endocrine-only group (hazard ratio for invasive disease recurrence, new primary cancer [breast cancer or another type], or death, 1.02; 95% CI, 0.82 to 1.26; *P* = 0.89)^6^. In premenopausal women, the rate of iDFS at 5 years for the chemoendocrine group was 93.9% versus 89.0% for the endocrine-only group (absolute difference, 4.9 percentage points), with a significant benefit from the addition of chemotherapy to ET (hazard ratio for invasive disease recurrence, new primary cancer [breast cancer or another type], or death, 0.60; 95% CI, 0.43–0.83; *P* = 0.002). All subgroups had a greater iDFS benefit with chemoendocrine therapy compared to endocrine therapy only. The hazard ratios were similar regardless of the type of nodal sampling, number of positive nodes, and recurrence score (0–13 or 14–25)^6^. Several clinical trials are now prospectively testing the clinical utility of MRD monitoring and early intervention in HR+ (DARE; NCT04567420) and triple-negative (C-TRAK; NCT03145961) early-stage breast cancers. Despite these efforts, many questions on risk stratification in the early breast cancer setting remain to be answered. While multiparametric scores, including both clinicopathological and genomic variables, retain the highest prognostication validity, and testing tools have all been shown to accurately predict both relapse of disease and overall survival, it is unknown which one should be preferred. We need to routinely assess features of the disease as stage, biology and genomic profile to better quantify the risk of HR+/HER2- early-stage breast cancer recurrence. This assessment, may in the feature with the help of artificial intelligence tools or algorithms, we help us to identify those patients candidate to endocrine therapy or to chemotherapy followed by adjuvant CDK 4–6 inhibitors. Potential new technologies, such as liquid biopsy, need to be studied to assess their clinical utility and clinical validity for assessing the risk of relapse or to identify molecular residual disease (MRD) after surgery systemic therapy. We need also to better quantify the magnitude of clinical benefit derived from escalation with CDK 4/6 inhibitors in the context of the world setting and using patient-reported outcomes tools.

### Reporting summary

Further information on research design is available in the Nature Research Reporting Summary linked to this article.

## Supplementary information

Reporting Summary

## Data Availability

Source data for all figures and tables are provided in the paper. No new data sets have been generated or analyzed for this article.
